# Effects of short-term bisoprolol on perioperative myocardial injury in patients undergoing non-cardiac surgery: a randomized control study

**DOI:** 10.1038/s41598-021-01365-5

**Published:** 2021-11-10

**Authors:** Wanwarang Wongcharoen, Thanyalak Chotayaporn, Kavint Chutikhongchalermroj, Apichat Tantraworasin, Somcharoen Saeteng, Supapong Arworn, Kittipan Rerkasem, Arintaya Phrommintikul

**Affiliations:** 1 Division of Cardiology, Department of Internal Medicine, Faculty of Medicine, Chiang Mai University, Chiang Mai, Thailand; 2 Division of Thoracic Surgery, Department of Surgery, Faculty of Medicine, Chiang Mai University, Chiang Mai, Thailand; 3 Division of Vascular Surgery, Department of Surgery, Faculty of Medicine, Chiang Mai University, Chiang Mai, Thailand; 4 Environmental Occupational Health Sciences and Non Communicable Diseases Center of Excellence, Research Institute of Health Sciences, Chiang Mai University, Chiang Mai, Thailand; 5 Center for Medical Excellence, Faculty of Medicine, Chiang Mai University, Chiang Mai, Thailand

**Keywords:** Cardiology, Medical research

## Abstract

The protective role of preoperative beta-blocker in patients undergoing non-cardiac surgery is unknown. We aimed to evaluate the effects of beta-blocker on perioperative myocardial injury in patients undergoing non-cardiac surgery. We consecutively enrolled 112 patients undergoing non-cardiac surgery. They were randomly allocated to receive bisoprolol or placebo given at least 2 days preoperatively and continued until 30 days after surgery. The primary outcome was incidence of perioperative myocardial injury defined by a rise of high-sensitive troponin-T (hs-TnT) more than 99th percentile of upper reference limit or a rise of hs-TnT more than 20% if baseline level is abnormal. Baseline characteristics were comparable between bisoprolol and placebo in randomized cohort Mean age was 62.5 ± 11.8 years and 76 (67.8%) of 112 patients were male. Among 112 patients, 49 (43.8%) underwent vascular surgery and 63 (56.2%) underwent thoracic surgery. The median duration of assigned treatment prior to surgery was 4 days (2–6 days). We did not demonstrate the significant difference in the incidence of perioperative myocardial injury [52.6% (30 of 57 patients) vs. 49.1% (27 of 55 patients), P = 0.706]. In addition, the incidence of intraoperative hypotension was higher in bisoprolol group than placebo group in patients undergoing non-cardiac surgery [70.2% (40 of 57 patients) vs. 47.3% (26 of 55 patients), P = 0.017]. We demonstrated that there was no statistically significant difference in perioperative myocardial injury observed between patients receiving bisoprolol and placebo who had undergone non-cardiac surgery.

## Introduction

Perioperative cardiac complications are of significant concern in patients undergoing non-cardiac surgery^1^. It has been shown that cardiac troponins have a high sensitivity and specificity in detecting myocardial injury. The prevalence of perioperative myocardial injury evaluated by high-sensitive troponin T (hs-TnT) was reported to be as high as 45% in patients undergoing non-cardiac surgery^2^. Previous studies have demonstrated that an elevated postoperative cardiac troponin level, irrespective of clinical symptoms, is associated with high risk of short-term and long-term major cardiovascular events and cardiovascular death^3^. Therefore, prevention of a perioperative myocardial injury is essential to improve the overall postoperative outcomes.

Beta-blocker has been described as a potential measure to prevent perioperative myocardial injury. Previous animal study has shown that early administration of metoprolol after ST-segment elevation myocardial infarction (STEMI) could diminish the progression of ischemic injury^4^. This finding was confirmed in randomized human study which demonstrated that intravenous metoprolol administration in patients with anterior STEMI improved ECG markers of myocardial ischemia before reperfusion^5^. The precise mechanism by which beta-blocker decreases perioperative myocardial ischemia has been proposed. Beta-blocker improves oxygen balance of myocardium by negative inotropic effect and the reduction in blood pressure and heart rate. Furthermore, beta-blocker may attenuate the risk for plaque rupture by decreasing circumferential stress on the atherosclerotic plaques^6,7^. Previous study have shown that patients receiving bisoprolol at least 7 days before the intermediate-risk surgery had a significant decrease in 30-day nonfatal myocardial infarction and cardiovascular mortality^8^. However, POISE study has shown that perioperative high-dose metoprolol beginning within 24 h prior to non-cardiac surgery is associated with the increased risk of bradycardia, hypotension, stroke and mortality^9^. With this regard, international guidelines state that beta-blocker treatment is not advised to start within one day before the surgery^10,11^. The optimal duration of beta-blockers given before the surgery and their benefits, in patients at intermediate or high surgery risk, have not been clearly elucidated. It has been suggested by the guidelines that starting beta-blocker more than 1 day may be considered^10,11^, despite the unclear benefit of short-term of beta-blocker^12,13^. Previous cohort studies have shown that short-term beta-blocker given less than 1 week before surgery may be harmful^12,13^. Nevertheless, no randomized control trials have been conducted to clarify this issue. The European perioperative guideline suggested bisoprolol or atenolol if beta-blocker is considered before the surgery based on observational studies^10,14^. Therefore, we aimed to investigate whether the short-term oral bisoprolol given at least 2 days preoperatively could prevent myocardial injury after surgery in patients undergoing non-cardiac surgery as compared to placebo.

## Materials and methods

### Study populations

**Figure 1:**
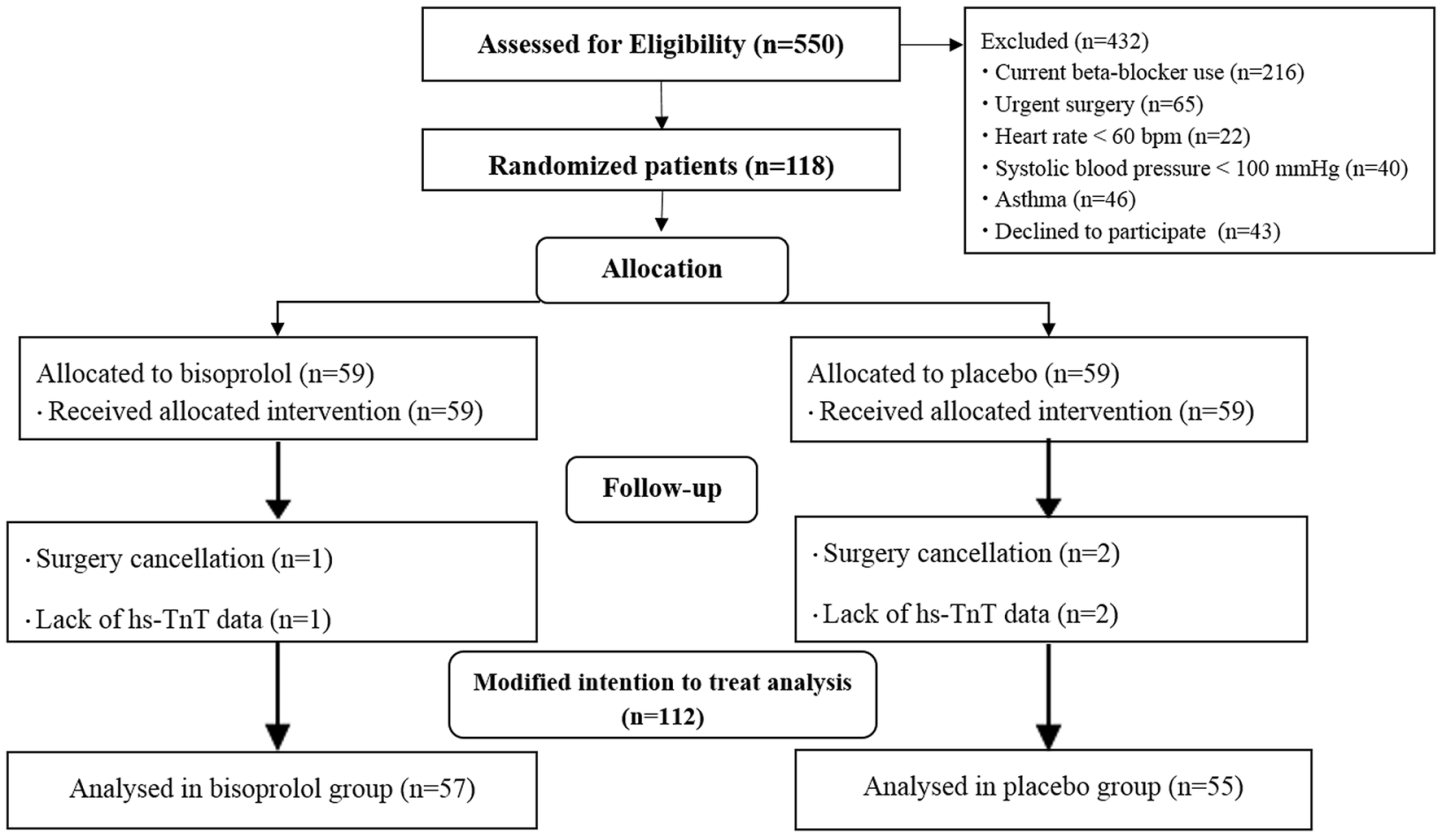
CONSORT flow diagram of randomized patient.

The present study was a randomized, prospective, double-blinded, placebo-controlled trial performed at Maharaj Nakorn Chiang Mai Hospital, Chiang Mai University. The study was approved by the ethics committee of the Faculty of Medicine, Chiang Mai University (MED-2558-03128) and registered on 29/03/2018 to http://www.clinicaltrials.in.th/ which submission number TCTR20180329005. The investigations were carried out in accordance with the Declaration of Helsinki, including written informed consent of all participants. During a period of November 2012 to January 2017, we had prospectively enrolled 112 consecutive patients, aged more than 18 years (Fig. 1), who underwent elective non-cardiac thoracic or vascular surgery. Informed consent was attained from each patient to take part in the study. The treatment allocation was done by block of four-randomization. To ensure the blinding situation during the trial, the research assistance performed randomization and assigned patients to intervention. The primary care team including surgeons, anesthesiologists, nurses; and patients were blinded from study treatment allocation. The medication was delivered to the patients by the blinded nurses. The outcome of the studies were assessed by the investigators who were unaware of the assigned treatment. All the biomarkers were tested immediately after sampling. The results of all the biomarkers were reported to the primary care surgeons. The management of the myocardial infarction and stroke after surgery were left at the discretion of the primary care team.

Bisoprolol tablets were purchased from Maharaj Nakorn Chiang Mai hospital. Placebo tablets were provided by Department of Pharmaceutical Sciences, Faculty of Pharmacy, Chiang Mai University. One bisoprolol tablet contained 2.5 mg of bisoprolol. The placebo tablet was made from maize starch with comparable appearance to bisoprolol tablet.

The enrolled patients were randomly assigned to receive bisoprolol or placebo given at least 2 days preoperatively and continued until 30 days after surgery. The patients were excluded if they had been taking beta-blockers, had contraindication of the use of beta-blockers including asthma, acute decompensated heart failure, second or third degree AV block, had baseline resting heart rate (HR) < 60 beats per minute (bpm), had baseline systolic blood pressure (SBP) < 100 mmHg, had plan to use beta–blocker before surgery or during the first 30 postoperative days for other reasons, had concurrent use of verapamil, diltiazem or amiodarone, urgent or emergency surgery within 24 h, refused to participate in the randomized study.

Bisoprolol was started at the dose of 2.5 mg orally per day (1.25 mg per day if left ventricular ejection fraction was less than 40%)^15^. During hospitalization, resting heart rate was daily evaluated and the dose of assigned drug was adjusted with stages of 1.25 or 2.5 mg per day. The maximum dose was 10 mg per day, targeting at a heart rate of 50 to 70 bpm. The same dose of studied drug was maintained if HR 50–69 bpm and/or SBP 90–99 mmHg. A 1.25 mg of studied drug was added if HR 70–80 bpm and/or SBP 100–119 mmHg. A 2.5 mg of studied drug was added if HR ≥ 81 bpm and SBP ≥ 120 mmHg. The administration of studied drug was temporarily stopped if any of the following occurred; systolic blood pressure less than 90 mmHg, resting heart rate less than 50 bpm, heart failure or bronchospasm, PR interval ≥ 0.30 s, second or third degree atrioventricular block.

An electrocardiograph (ECG) (HP M1700A, Hewlett Packard, Palo Alto, CA, USA) was recorded before surgery and on the first, second, and third and 30th days after surgery. The hs-TnT level was measured before surgery and on the first, second, and third days after surgery. The level of N-terminal-pro B-type natriuretic peptide (NT-proBNP) and high-sensitive C-reactive protein (hs-CRP) was also assessed before and after surgery. An echocardiography (Phillips Sonos 7500 Ultrosounds) was performed at baseline before surgery to evaluate left ventricular ejection fraction (LVEF). LVEF were measured using modified biplane Simpson’s methods. NT-proBNP and hs-TnT were evaluated with electrochemiluminescence immunoassay by using the Cobas e601 system (Roche Diagnostics). The detection limit of hs-TnT was 3 ng/L, a cut-off point at 99th percentile was 14 ng/L, and a coefficient of variation of less than 10% was at 13 ng/L. The analytical measurement range of NT-proBNP was 5–35,000 pg/mL. Hs-CRP was assessed with particle-enhanced immunoturbidimetric assay by using the Cobas c502 system (Roche Diagnostic) with an analytical measurement range of 0.15–20 mg/L.

The outcomes were determined by 2 independent cardiologists who were blinded to treatment allocation. The study protocol was approved by Institutional Review Board of Faculty of Medicine, Chiang Mai University.

The primary endpoint of our study was the incidence of perioperative myocardial injury that occurred within 3 days after surgery. The secondary endpoints of the study were the incidence of perioperative myocardial infarction, postoperative stroke or all-cause death that occurred within 30 days after surgery.

### Definitions

- Perioperative myocardial injury was defined as a rise of hs-TnT more than the 99th percentile of the upper reference limit or a rise of hs-TnT more than 20% if baseline level is elevated (> 14 ng/L)^16^.
- The diagnosis of myocardial infarction required at least two of the followings^16^.
  1. Chest pain (angina symptom) lasting more than 20 min.
  2. ECG alterations including acute ST-segment elevation followed by appearance of Q waves or loss of R waves, or new left bundle branch block, or new ST segment depression, or new persistent T wave inversion for at least 24 h, which sustains for at least 24 h.
  3. A positive hs-TnT measurement with characteristic rise and fall.
- Non-cardiac thoracic surgery is defined as the procedure to diagnosis and therapy of the diseased conditions of the trachea, lungs, esophagus and mediastinum include by thoracotomy or video-assisted thoracic surgery.
- Non-cardiac vascular surgery is defined as the vascular surgery including percutaneous vascular intervention except arteriovenous shunt and vein stripping procedures.
- Revised cardiac risk index comprised five variables including the history of coronary artery disease, heart failure, diabetes currently taking insulin therapy, preoperative serum creatinine more than 2.0 mg/dL or glomerular filtration rate less than 60 mL/min and history of transient ischemic attack or stroke^10,11,17^.
- Intraoperative hypotension is defined as systolic blood pressure less than 90 mmHg during surgery^18,19^.

### Statistical analysis

All analyses were done on the modified intention to treat basis, excluding the randomized patients who neither underwent surgery nor had hs-TnT levels after surgery. Demographic and perioperative variables were compared between groups with a t test for normally distributed values; otherwise, the Mann–Whitney U test was used. Proportions were compared by Chi-square test or Fisher exact test when appropriate. Repeated measures analysis of variance was used to compare the differences of hs-TnT level, heart rate and blood pressure before and after surgery. Continuous variables with normal distribution were presented as means ± standard deviation. Results with non-normal distribution are expressed as median ± interquartile range. The Kolmogorov–Smirnov test was used to assess normality of the data. Categorical variables were displayed as percentages. A probability values < 0.05 (2-tailed) was considered significant. Hazard ratios (HRs) and 95% confidence intervals (CIs) to assess the risk of the primary end point according to potential confounding variables were determined by logistic regression.

### Sample size calculation

We calculated the sample size at the power of 80% with level of statistical significant at 95%. The incidence of perioperative myocardial injury (hs-TnT ≥ 14 ng/L) was estimated at 50%^2^. We estimated a 50% relative risk reduction associated with bisoprolol therapy^8,20^. The estimated total sample size was 116 patients (58 patients in each group). We initially enrolled a total of 118 patients in the study. However, 6 patients were excluded due to the cancellation of surgery and the lack of hs-TnT data. In this regard, a total of 112 patients who had the preoperative and postoperative hs-TnT data were included in the final modified intention to treat analysis as mentioned above (Fig. 1).

## Results

We prospectively enrolled 112 consecutive patients who underwent elective non-cardiac surgery and met inclusion criteria in the present study. Baseline characteristics were comparable between bisoprolol and placebo, with respect to age, gender, comorbidities, concomitant medications, and type of surgery. The mean age was 62.5 ± 11.8 years in bisoprolol group and 64.4 ± 13.1 years in placebo group. The majority of patients (70.2%) group had no clinical risk factors according to revised cardiac risk index (RCRI). Only 7 (6.3%) patients had more than 1 RCRI (Tables 1, 2). Among 112 patients, 49 (43.8%) underwent vascular surgery and 63 (56.2%) underwent thoracic surgery. The patient’s risk status was also assessed by American Society of Anesthesiologists Physical Status Classification System (ASA scores). Majority of the patients in both groups had ASA score I and II, indicating the low-risk status of studied population (Table 1).

**Table 1:** Baseline characteristics between bisoprolol and placebo groups.

Characteristics	Bisoprolol (N = 57)	Placebo (N = 55)	P value
Age (years)	62.5 ± 11.8	64.4 ± 13.1	0.416
Male	37 (67.3%)	27 (50.9%)	0.177
BMI (kg/m^2^)	21.5 ± 4.3	21.3 ± 4.1	0.876
DM	10 (18.2%)	7 (13.2%)	0.600
DM requiring insulin therapy	5 (9.1%)	3 (5.7%)	0.716
Hypertension	30 (54.5%)	23 (43.4%)	0.256
History of stroke	3 (5.5%)	2 (3.8%)	1.000
Coronary artery disease	4 (7.0%)	4 (7.3%)	1.000
Heart failure	0 (0%)	0 (0%)	
LVEF (%)	64.7 ± 8.3	67.3 ± 8.1	0.104
**ASA score**			
ASA I	24 (42.1%)	30 (54.5%)	0.407
ASA II	26 (45.6%)	19 (34.5%)	
ASA III	7 (12.3%)	6 (10.9%)	
Hemoglobin (gm/dL)	11.8 ± 1.8	12.2 ± 2.5	0.411
Creatinine (mg/dL)	1.2 ± 1.2	1.1 ± 1.2	0.616
Antiplatelet	15 (27.3%)	10 (18.9%)	0.364
Statin	17 (30.9%)	12 (22.6%)	0.389
ACEI/ARB	6 (10.9%)	6 (11.3%)	1.000
Median duration of preoperative assigned therapy (days)	3 ± 4	4 ± 2	0.669

*ACEI/ARB* angiotensin converting enzyme inhibitor/Angiotensin receptor blocker, *BMI* body mass index, *DM* diabetes mellitus, *hs-TNT* high-sensitive troponin-T, *LVEF* left ventricular ejection fraction.

**Table 2:** Perioperative features between bisoprolol and placebo groups.

Perioperative features	Bisoprolol (N = 57)	Placebo (N = 55)	P value
**Thoracic surgery**	32 (56.1%)	31 (56.4%)	1.000
Thoracotomy/lobectomy	19 (33.3%)	15 (27.3%)	
Video-assisted thoracoscopic surgery	13 (22.8%)	16 (29.1%)	
**Vascular surgery**	25 (43.9%)	24 (43.6%)	1.000
Artery bypass graft	9 (15.8%)	10 (18.2%)	
Abdominal aortic aneurysm repair	9 (15.8%)	6 (10.9%)	
Percutaneous transluminal angioplasty	5 (8.8%)	6 (10.9%)	
**Anesthesia**			
General	56 (98.2%)	52 (94.5%)	0.395
Epidural or spinal	1 (1.8%)	3 (5.5%)	
Preoperative hs-TNT (ng/L)	9.5 ± 10.6	9.5 ± 11.1	0.796
Postoperative peak hs-TNT (ng/L)	18.8 ± 19.6	15.7 ± 19.1	0.928
Preoperative NT-proBNP (ng/L)	122.6 ± 257.2	128.8 ± 270.6	0.571
Postoperative NT-proBNP (ng/L)	586.2 ± 703.0	370.0 ± 809.6	0.752
Preoperative hs-CRP (mg/L)	3.6 ± 25.0	4.2 ± 15.5	0.581
Postoperative hs-CRP (mg/L)	135.0 ± 100.1	111.0 ± 75.0	0.158
Preoperative creatinine (mg/dL)	1.2 ± 1.17	1.19 ± 1.16	0.616
Postoperative creatinine (mg/dL)	1.2 ± 1.4	1.0 ± 0.5	0.844
RCRI 0	40 (70.2%)	37 (67.3%)	0.906
RCRI 1	13 (22.8%)	15 (27.3%)	
RCRI 2	2 (3.5%)	2 (3.6%)	
RCRI 3	2 (3.5%)	1 (1.8%)	

*hs-CRP* high-sensitive C-reactive protein, *hs-TnT* high-sensitive troponin-T, *NT-proBNP* N-terminal-pro B-type natriuretic peptide, *RCRI* revised cardiac risk index.

**Figure 2:**
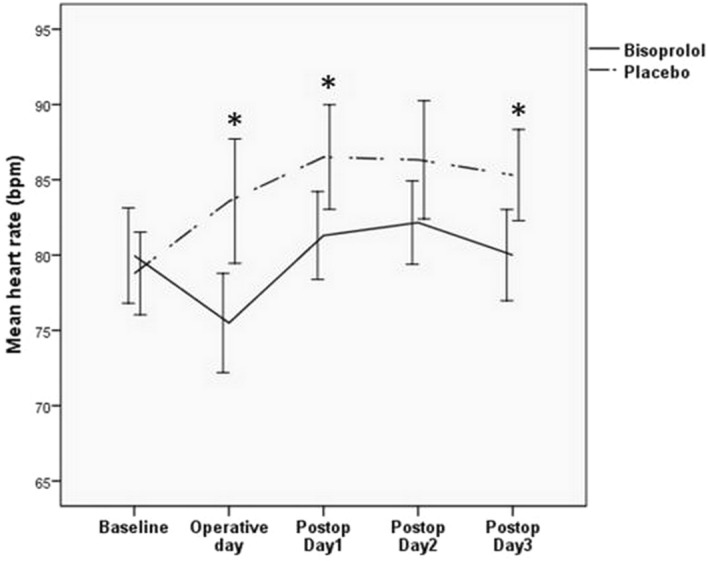
The effect of bisoprolol on heart rate during perioperative period compared to placebo. Among patient receiving bisoprolol (solid line), the mean heart rate was significantly reduced on operative day compared to baseline. It then significantly increased after surgery. On the contrary, in patients receiving placebo (dashed line), the mean heart rate increased on the operative day, the continued to increase after surgery. The heart rate on operative day and postoperative day 1–3 was significantly lower in bisoprolol group. *Statistically significant P < 0.05, compared to placebo.

The median duration of preoperative bisoprolol and placebo therapy was 3 ± 4 days and 4 ± 2 days, respectively (P = 0.669). The mean dose of bisoprolol before surgery was 4.7 ± 2.6 mg. Among patients receiving bisoprolol, the mean heart rate decreased from 80 ± 12 bpm at baseline to 75 ± 12 bpm on operative day. It then increased to 81 ± 11 bpm, 82 ± 10 bpm and 80 ± 11 bpm on the first, second and third day after surgery (P = 0.008). On the contrary, in patients receiving placebo, the mean heart rate increased from 79 ± 10 bpm at baseline to 83 ± 15 bpm on the operative day, then continued to increase to 86 ± 13 bpm, 86 ± 15 bpm and 85 ± 11 bpm on the first, second and third day after surgery (P = 0.003). Compared between bisoprolol and placebo, the heart rate on operative day and postoperative day 1–3 was significantly lower in bisoprolol group (Fig. 2).

**Figure 3:**
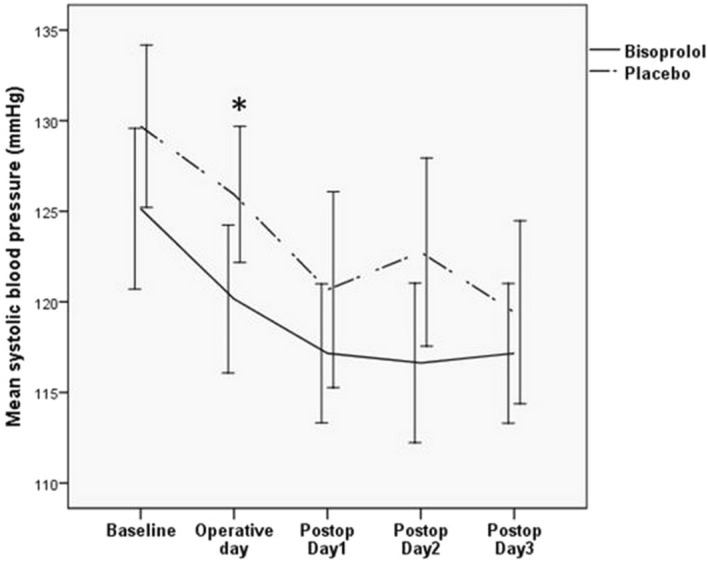
The effects of bisoprolol on systolic blood pressure during perioperative period compared to placebo. The systolic blood pressure was significantly reduced after surgery compared to baseline in both bisoprolol (solid line), and placebo (dashed line) groups. When compared between 2 groups, the systolic blood pressure on operative day was significantly lower in bisoprolol compared to placebo. *Statistically significant P < 0.05, compared to placebo.

The significantly lower systolic blood pressure after surgery compared to baseline was observed in both bisoprolol and placebo groups. Among patients receiving bisoprolol, the mean systolic blood pressure decreased from 125 ± 17 mmHg at baseline to 120 ± 15 mmHg on operative day. It then continued to decrease to 117 ± 14 mmHg, 117 ± 16 mmHg and 117 ± 14 mmHg on the first, second and third day after surgery (P < 0.001). Similarly, in patients receiving placebo, the mean systolic blood pressure decreased from 130 ± 17 mmHg at baseline to 126 ± 14 mmHg on operative day. It then decreased to 121 ± 20 mmHg, 123 ± 19 mmHg and 119 ± 18 mmHg on the first, second and third day after surgery (P < 0.001).When compared between 2 groups, the systolic blood pressure on operative day was significantly lower in bisoprolol compared to placebo (Fig. 3).

**Figure 4:**
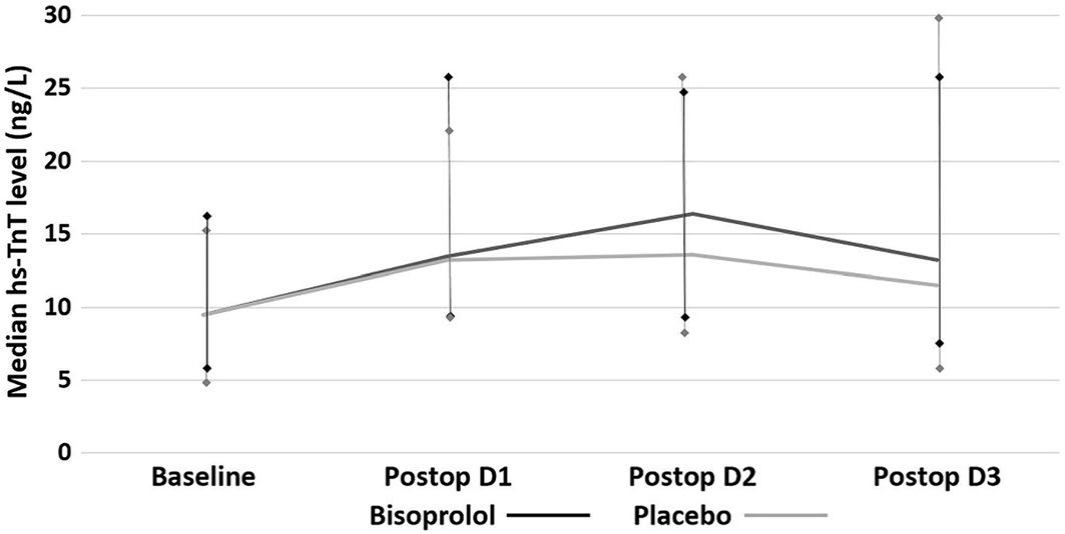
The effect of bisoprolol on median level of hs-TnT after surgery compared to placebo. There was no difference of median level of hs-TnT after surgery between bisoprolol (black line) and placebo (grey line) groups.

We demonstrated that incidence of perioperative myocardial injury did not differ between patients receiving bisoprolol and placebo [52.6% (30 of 57 patients) vs. 49.1% (27 of 55 patients), P = 0.706, respectively]. Figure 4 shows the median hs-TnT level before and after surgery between bisoprolol and placebo groups. The median postoperative hs-TnT level was not different between 2 groups. Among 31 patients with elevated baseline hs-TnT level, 9 of 15 (60%) patients in bisoprolol group and 12 of 16 (75%) patients in placebo group developed perioperative myocardial injury (P = 0.458). Of 81 patients with normal baseline hs-TnT level, there was also no difference of perioperative myocardial injury between bisoprolol and placebo groups [50.0% (21 of 42 patients) vs. 38.5% (15 of 39 patients), P = 0.372, respectively]. Four of 57 patients in bisoprolol group and two of 55 patients in placebo group developed perioperative myocardial infarction (7.0% vs. 3.6%, P = 0.679). Only one patient in bisoprolol group died during hospitalization due to sepsis. No stroke occurred in both groups of patients. In addition, preoperative and postoperative levels of hs-TnT, hs-CRP and NT-BNP were comparable between bisoprolol and placebo groups.

Incidence of serious adverse events and drug discontinuation was not different between bisoprolol and placebo groups (Table 3). However, the incidence of intraoperative hypotension was significantly higher in bisoprolol group than placebo group [70.2% (40 of 57 patients) vs. 47.3% (26 of 55 patients), P = 0.017].

**Table 3:** Adverse events in bisoprolol and placebo groups.

Baseline characteristics	Bisoprolol (N = 57)	Placebo (N = 55)	P value
Bronchospasm	1 (1.8%)	5 (9.1%)	0.110
Heart failure	2 (3.5%)	4 (7.3%)	0.434
Atrial fibrillation	2 (3.5%)	2 (3.6%)	1.000
Postoperative stroke	0 (0%)	0 (0%)	
Postoperative sepsis	4 (7.0%)	4 (7.3%)	1.000
Postoperative myocardial infarction	4 (7.0%)	2 (3.6%)	0.679
Intraoperative hypotension	40 (72.7%)	26 (49.1%)	0.017
Postoperative hypotension	10 (17.5%)	14 (25.5%)	0.361
Postoperative bradycardia	3 (5.3%)	2 (3.6%)	1.000

## Discussion

Perioperative cardiac complications increase the risk of short-term and long-term morbidity and mortality in patients undergoing non-cardiac surgery and remain a significant concern in this group of patients. Nevertheless, the detection of myocardial infarction occurring after surgery has become a challenge due to the presence of postoperative analgesic agent that may obscure the ischemic symptoms. In addition, the high prevalence of ECG abnormalities in patients after non-cardiac surgery has made ECG findings less specific in the diagnosis of myocardial infarction. Several investigators have shown that the elevation of perioperative troponin level, irrespective of symptoms, is associated with increased risk of death at 30 days^21^. As a result, perioperative myocardial injury detected by the elevation of perioperative troponin level can be a predictor of adverse postoperative outcomes.

POISE study has shown that beta blocker started within 24 h or less before non-cardiac surgery decreases risk of nonfatal myocardial infarction but increases risk of bradycardia, hypotension, stroke, and mortality^9^. Perioperative mortality in patients randomized to metoprolol succinate was associated with perioperative bradycardia, hypotension, and stroke. The use of a high dose of metoprolol without dose titration in POISE study may lead to higher risk of perioperative hypotension. On the contrary, DECREASE-IV study has demonstrated that the patients receiving bisoprolol at least 7 days before surgery (an average of 34 days before surgery) had a significant reduction in 30-day cardiovascular death and nonfatal myocardial infarction^8^. However, the validity of the results from DECREASE trials led by Poldermans has been scrutinized because of concerns about scientific misconduct.

The meta-analysis by Bouri et al. which excluded all DECREASE trials showed the significant 27% increase in mortality from the initiation of perioperative beta-blocker^22^. However, all the trials in this meta-analysis initiated beta-blocker within 1 day or less prior to surgery. As the evidence pointed out toward the harmful effect of high-dose beta-blocker started within 24 h or less before the surgery, beta-blocker started within 24 h before non-cardiac surgery is discouraged by international guidelines^10,11^.

A series of meta-analyses showed the conflicting outcomes on the safety and efficacy of pre-operative beta-blockers^7,22–24^. Nevertheless, their findings were relatively consistent in demonstrating that the use of perioperative beta-blockers had significant harmful associations with bradycardia, hypotension and stroke. However, they could reduce perioperative myocardial infarction. The systematic review by Wijeysundera and colleagues demonstrated that without the controversial DECREASE studies, there were insufficient data on beta-blocker started 2 or more days prior to surgery^7^. With this regard, the 2014 AHA/ACC guidelines have stated that it may be reasonable to begin perioperative beta-blockers long enough in advance to assess safety and tolerability, preferably more than 1 day before surgery as a class IIb indication. In addition, they have stated that in patients with intermediate- or high-risk preoperative tests, it may be reasonable to begin beta-blockers as a class IIb indication. They concluded that randomized control trials were needed to address this knowledge gap^7,11^. Aside from the controversial DECREASE trials, to the best of our knowledge, our study is the first randomized controlled trial to examine the effect of beta-blocker given more than 1 day before surgery in patients undergoing non-cardiac thoracic and vascular surgery.

In the present study, we demonstrated that there was no statistically significant difference in perioperative myocardial injury and myocardial infarction between patients receiving short-term beta-blocker and those receiving placebo who had undergone non-cardiac surgery. Of importance, we observed the higher incidence of intraoperative hypotension in bisoprolol group. This may suggest the possible harmful effect of short-term beta-blocker, similar to those observed in the POISE study^9^.

At the time of the enrollment, the international guidelines stated that perioperative beta-blocker was discouraged in patients undergoing the low-risk surgery. Therefore, in this study, we enrolled only patients scheduled for elective non-cardiac thoracic and vascular surgery which was considered as the intermediate- and high-risk surgery. We did not exclude the low-risk patients with 0 revised cardiac risk index from our study due to the fact that the effect of perioperative beta-blocker in low-risk population had not been elucidated at the time of study start.

The majority of patients in our study had no clinical risk factors regarding revised cardiac risk index. Results of our study are in accordance with the observational data obtained by Jørgensen et al. The investigators have demonstrated the possible harmful effect of perioperative beta-blocker in patients with 0–1 RCRI^25,26^. In this regard, our findings reinforce the possible harmful effect of perioperative beta-blocker in low-risk patients, irrespective of the risk of surgery.

Nevertheless, the potential benefit of longer duration of beta-blocker and in higher-risk population is still unknown. The preventive effect of beta-blocker on the coronary plaque rupture may require some times to develop. It has been described that prolonged beta-blocker treatment decreases inflammatory cytokines and impedes coronary atherosclerosis progression. Previous study has demonstrated the preferential neutrophil stunning by metoprolol which may reduce coronary microvascular obstruction^27^. As a result, the duration of preoperative beta-blocker may affect the postoperative outcomes.

The different beta-blockers may have different effects on perioperative myocardial injury. Previous animal study demonstrated that only metoprolol, but not atenolol or propranolol, reduced infarct size and inflammation a rat model of MI^28^. Furthermore, beta-blockers which exert ancillary cardioprotective properties such as carvedilol through alpha 1-adrenoceptor blockade and antineutrophil and antioxidative actions, or nebivolol which enhances nitric oxide signalling may have additional benefit on cardioprotection^29^. Importantly, the interference of co-morbidities and co-medications with cardioprotection has been established^30^. Therefore, these confounders of cardioprotection may attenuate the potential benefit of beta-blocker in patients undergoing non-cardiac injury.

In addition, it has been demonstrated that discontinuation of beta-blocker treatment early after surgery correlates with higher risk of 1-year death compared with patients not using beta-blockers^31^. Therefore, it is crucial to continue beta-blocker in patients with pre-existing beta-blocker therapy.

The major limitation of the present study was the small sample size of the studied population and the inadequate statistical power to exclude a Type II error. The calculated sample size of 116 patients was insufficient because we overestimated the benefit of preoperative bisoprolol, based on DECREASE trials which are currently considered as the scientific misconduct studies^8,20^. The sample size of nearly 4000 patients are needed to declare the difference between 2 groups with adequate statistical power. Therefore, the results of the study should be interpreted with caution. In addition, our studied population had high baseline hs-TnT, NT-proBNP and hs-CRP despite the relatively low RCRI. The high level of these biomarkers usually indicate the high risk population with multiple co-morbidities. It is possible that the co-morbidities in our patients might have been underreported or underdiagnosed. Also, the interpretation of changes in troponin levels in the post-operative period, can be quite challenging such as presence of renal dysfunction after surgery. Furthermore, it is not clear whether the effect of bisoprolol used in our studied population can be applied to other beta blockers.

## Conclusions

We demonstrated that there was no statistically significant difference in perioperative myocardial injury observed between patients receiving bisoprolol and placebo who had undergone non-cardiac thoracic or vascular surgery. In addition, the incidence of intraoperative hypotension was higher in bisoprolol group than placebo group in patients undergoing non-cardiac surgery.

## Data Availability

The informed consent given by Effects of short-term bisoprolol on perioperative myocardial injury in patients undergoing non-cardiac surgery: a randomized control study does not cover data posting in public databases. However, data are available upon request from Effects of short-term bisoprolol on perioperative myocardial injury in patients undergoing non-cardiac surgery: a randomized control study should be sent to arintaya.p@cmu.ac.th and are subject to approval by the Faculty of Medicine, Chiang Mai University Ethics Committee.
